# Cold atmospheric plasma and adaptive antitumor immunity: current evidence with emphasis on T-cell responses

**DOI:** 10.3389/fimmu.2026.1935730

**Published:** 2026-10-28

**Authors:** Fawad Khan, Michael P. Schön

**Affiliations:** Department of Dermatology, Venereology and Allergology, University Medical Center Göttingen, Göttingen, Germany

**Keywords:** adaptive antitumor immunity, antigen presenting cells, cold atmospheric plasma, immune checkpoint blockade, immunogenic cell death, immunomodulation, plasma medicine, reactive oxygen and nitrogen species

## Abstract

Cold atmospheric plasma (CAP) is a redox-based technology that generates reactive oxygen and nitrogen species (RONS) and is being investigated as a potential immunomodulatory approach for cancer therapy and other clinical indications. This narrative review evaluates current evidence concerning CAP-mediated regulation of adaptive antitumor immunity, with particular emphasis on T-cell responses, immunogenic cell death (ICD), antigen-presenting cell (APC) activation, tumor microenvironment (TME) remodeling, and combination treatment with immune checkpoint blockade. Available studies indicate that CAP influences T-cell immunity predominantly indirectly by inducing tumor cell stress and death, the release of damage-associated molecular patterns (DAMPs), increasing antigen availability, dendritic cell (DC) activation, and remodeling of cytokine and chemokine networks. Some studies also report direct effects of carefully dosed CAP exposure on primary T cells; however, these findings remain limited and must be balanced against the substantial oxidative sensitivity of resting lymphocytes. Calreticulin exposure, endoplasmic reticulum stress, mitochondrial oxidative responses, extracellular ATP, and HMGB1 release have been reported in CAP-treated tumor models and are compatible with ICD, but these markers alone do not establish functional antitumor immunogenicity. Stronger evidence for functionally relevant adaptive antitumor immunity includes functional antigen uptake and presentation by DC, tumor-specific T-cell responses, evidence of adaptive immune-cell dependence, prophylactic vaccination protection, and resistance to tumor rechallenge. Preclinical studies further suggest that CAP or plasma-activated liquids can increase intratumoral CD4^+^ and CD8^+^ T-cell infiltration and improve responses to immune checkpoint blockade. Evidence concerning B-cell and antibody responses remains sparse. Clinical studies have primarily addressed the feasibility, safety, antimicrobial activity, or wound-healing effects of superficial cold plasma applications and should not be interpreted as clinical validation of CAP-based cancer immunotherapy. Translation will require standardized device parameters, gas composition, exposure dose, RONS dosimetry, delivery formats, and immune monitoring. Overall, CAP is a promising but incompletely validated platform for converting localized redox effects into therapeutically useful adaptive immune responses.

## Introduction

Immunotherapy has transformed the treatment landscape for cancer and chronic infections. However, durable clinical responses remain limited to a minority of patients receiving current immunotherapeutic agents, including immune checkpoint inhibitors and adoptive cellular therapies (1). Resistance mechanisms often emerge due to inadequate tumor immunogenicity, immunosuppressive microenvironments, active immune escape mechanisms, or dysfunctional innate signaling (2). Therefore, strategies that locally enhance tumor immunogenicity and improve T-cell access to tumor tissue remain of substantial interest.

Cold atmospheric plasma (CAP) is a near-room-temperature, partially ionized gas that generates a complex mixture of reactive oxygen and nitrogen species (RONS), electric fields, charged particles, and ultraviolet (UV) photons, allowing biological effects without substantial thermal damage to tissue (**Figure 1**). Depending on the plasma source, working gas, power, treatment distance, exposure time, target geometry, and composition of the treated liquid or tissue, CAP can induce distinct biological effects ranging from transient redox signaling to cytotoxic oxidative stress. CAP has established applications in surface decontamination and wound management and is increasingly being investigated in oncology because of its potential to preferentially damage tumor cells and modulate the local immune microenvironment (3–5).

**Figure 1:**
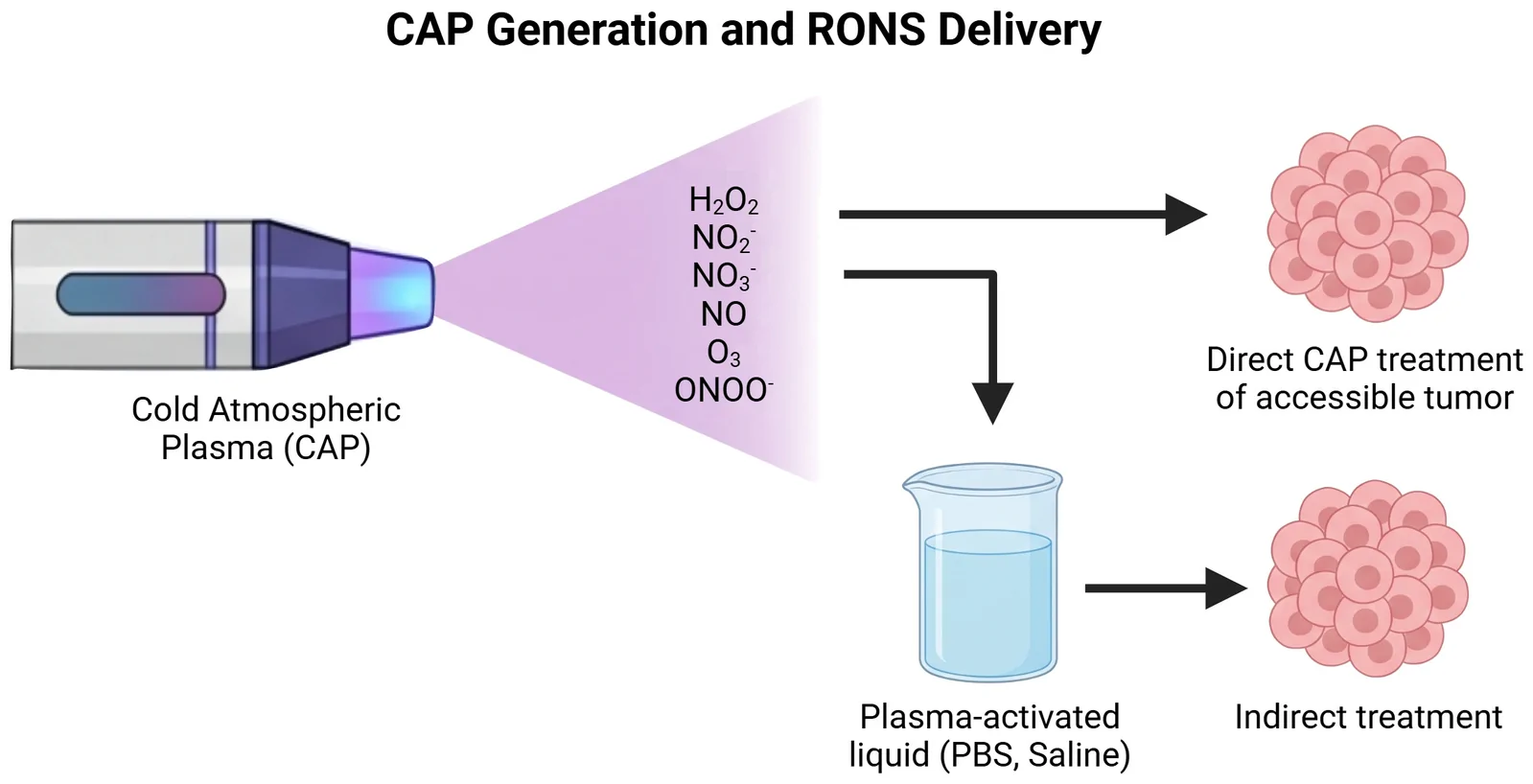
Cold atmospheric plasma generation and RONS delivery to tumor cells. Cold atmospheric plasma (CAP) can be applied directly to tumor cells or accessible tumor tissue, or indirectly through plasma-activated liquids, such as culture medium, saline, or hydrogel formulations. CAP-associated RONS, including H_2_O_2_, nitrite (NO_2_^−^), nitrate (NO_3_^−^), nitric oxide (NO), ozone (O_3_), and peroxynitrite (ONOO^−^), may contribute to redox signaling, oxidative stress, and downstream biological effects. The RONS composition, concentration, persistence, and biological activity depend on the plasma source, working gas, electrical settings, treatment geometry, exposure duration, target composition, and local tissue or liquid environment. Accordingly, CAP should not be regarded as a uniform intervention.

Available evidence indicates that rather than directly or uniformly activating T cells, CAP appears to promote adaptive antitumor immunity primarily by conditioning tumor cells and remodeling the tumor microenvironment (TME), thereby creating conditions favorable for T-cell priming and activation. CAP-induced oxidative stress may promote tumor cell death, increase antigen availability, modify tumor-associated proteins, induce immunogenic cell death (ICD)-associated danger signals, activate antigen-presenting cells (APCs), and alter local cytokine and chemokine networks (6–9). These events may enhance tumor antigen uptake, cross-presentation, T-cell priming, and intratumoral recruitment of effector T cells. However, the strength of evidence varies substantially among these proposed mechanisms. Evidence concerning B-cell and humoral responses is discussed separately as an important but underdeveloped area of research.

Previous reviews have addressed plasma medicine, CAP-induced ICD, CAP-mediated immunomodulation, the cancer immunity cycle, and the anticancer applications of CAP (4, 10–12). In particular, Živanić et al. reviewed CAP in relation to the cancer-immunity cycle and discussed therapeutic relevance and hydrogel-based delivery strategies (10). The present review focuses specifically on adaptive antitumor immunity, with emphasis on the quality, functional relevance, and mechanistic strength of evidence related to T cell-related outcomes. We evaluate the direct effects of CAP on lymphocytes as well as indirect mechanisms mediated by tumor cell stress, ICD-associated signaling, APC activation, oxidative modification of tumor-associated proteins, and TME remodeling. We further assess the evidence and limitations regarding tumor-specific T-cell priming, functional ICD, T-cell infiltration, memory, epitope spreading, and combination benefit with immune checkpoint blockade. More recent studies demonstrate that plasma-induced protein oxidation can enhance APC activation and that oxidized melanoma antigens can promote activation of selected cytotoxic T-cell subpopulations (13, 14). These findings provide a potential mechanistic link between CAP-derived redox chemistry and adaptive immune responses.

Accordingly, this review integrates evidence on ICD-associated signaling, APC activation, T-cell responses, oxidative antigen modification, immune-checkpoint modulation, and TME remodeling within a unified framework. We also identify key methodological and translational challenges, including the need for device-specific dosimetry, standardized characterization of RONS, robust functional immune endpoints, and clinical trials designed to evaluate antitumor outcomes.

Throughout this review, evidence is classified as (i) direct evidence of CAP effects on lymphocytes; (ii) indirect preclinical evidence mediated through tumor cell stress, ICD-associated signaling, APC activation, or TME remodeling; and (iii) proposed mechanisms or translational applications requiring further functional validation.

## Literature search strategy

A structured literature search was conducted in PubMed and Google Scholar for publications published from January 2010 through 9 October 2026, with particular emphasis on studies published from 2021 onward. The search was updated before resubmission to identify eligible publications. Search terms combined plasma-related terminology, including “cold atmospheric plasma”, “non-thermal plasma”, “gas plasma”, “plasma-activated medium”, and “plasma-activated liquid”, with cancer-immunology terms such as “cancer”, “tumor immunity”, “immunogenic cell death”, “T cells”, “B cells”, “immune checkpoint”, “tumor microenvironment”, “melanoma”, “radiotherapy”, “chemotherapy”, and “ROS/RNS”. Primary research articles, relevant systematic reviews, and selected mechanistic studies were included. Studies were prioritized when they provided information about plasma source, gas composition, treatment parameters, biological model, immune endpoints, or functional adoptive immune/antitumor outcomes. Additional studies were identified by manual screening reference lists from relevant articles.

## CAP effects on T cells: direct effects, indirect activation, and current evidence limits

RONS generated by CAP are important regulators of lymphocyte signaling, activation, differentiation, and cell death. Low and controlled oxidant signals may participate in physiological T-cell receptor signaling, whereas excessive or sustained oxidative stress can potentially impair proliferation, mitochondrial function, cytokine production, and viability (15–17). Consequently, CAP should not be regarded as a uniform immunomodulatory intervention. Its effects on T cells depend on the plasma source, working gas, electrical parameters, exposure duration, treatment distance, target medium, and the resulting short- and long-lived RONS.

Current evidence suggests that CAP affects T-cell responses through two principal routes: (i) direct exposure under carefully controlled low-dose conditions and, more commonly, (ii) indirect conditioning of the TME through induction of ICD, remodeling of cytokine networks, and activation of APCs.

## Direct CAP effects on T cells

At present, evidence for direct T-cell activation is limited. A small number of studies have reported that short, carefully dosed CAP exposure can modify cytokine production or inflammatory phenotypes in primary T cells and macrophages. *Ex vivo* CAP treatment of T cells followed by adoptive transfer into tumor-bearing mice demonstrated enhanced antitumor activity, potentially through increased production of proinflammatory cytokines and reduced expression of checkpoint-associated molecules (18). These findings suggest that, under certain conditions, direct plasma exposure may augment T-cell function. However, this possibility should be interpreted with caution because experimental conditions differ substantially across studies, and many reports involve mixed immune-cell populations rather than purified primary T-cell subsets.

Importantly, primary lymphocytes appear highly sensitive to CAP-derived oxidative stress. Experimental studies using peripheral blood mononuclear cell subsets, lymphocyte cell lines, and primary rat splenic lymphocytes have shown that direct plasma exposure can reduce lymphocyte viability and induce apoptosis, particularly at treatment intensities that are tolerated by some myeloid populations or tumor cells (19–22). Primary T cells may be more sensitive than transformed Jurkat cells (an immortalized human T cell line), emphasizing that findings obtained with immortalized cell lines should not be generalized to therapeutic T cells or endogenous tumor-reactive lymphocytes (20, 21).

Thus, direct CAP conditioning of T cells should currently be considered an experimental strategy, not an established approach for enhancing adoptive cell therapy (**Figure 2**). The available data do not yet define exposure windows that preserve T-cell viability while improving activation, proliferation, cytotoxicity, memory formation, metabolic fitness, trafficking, or resistance to exhaustion. Such studies will be essential before CAP can be considered for direct *ex vivo* conditioning of CAR-T cells, TILs, or engineered T-cell products.

**Figure 2:**
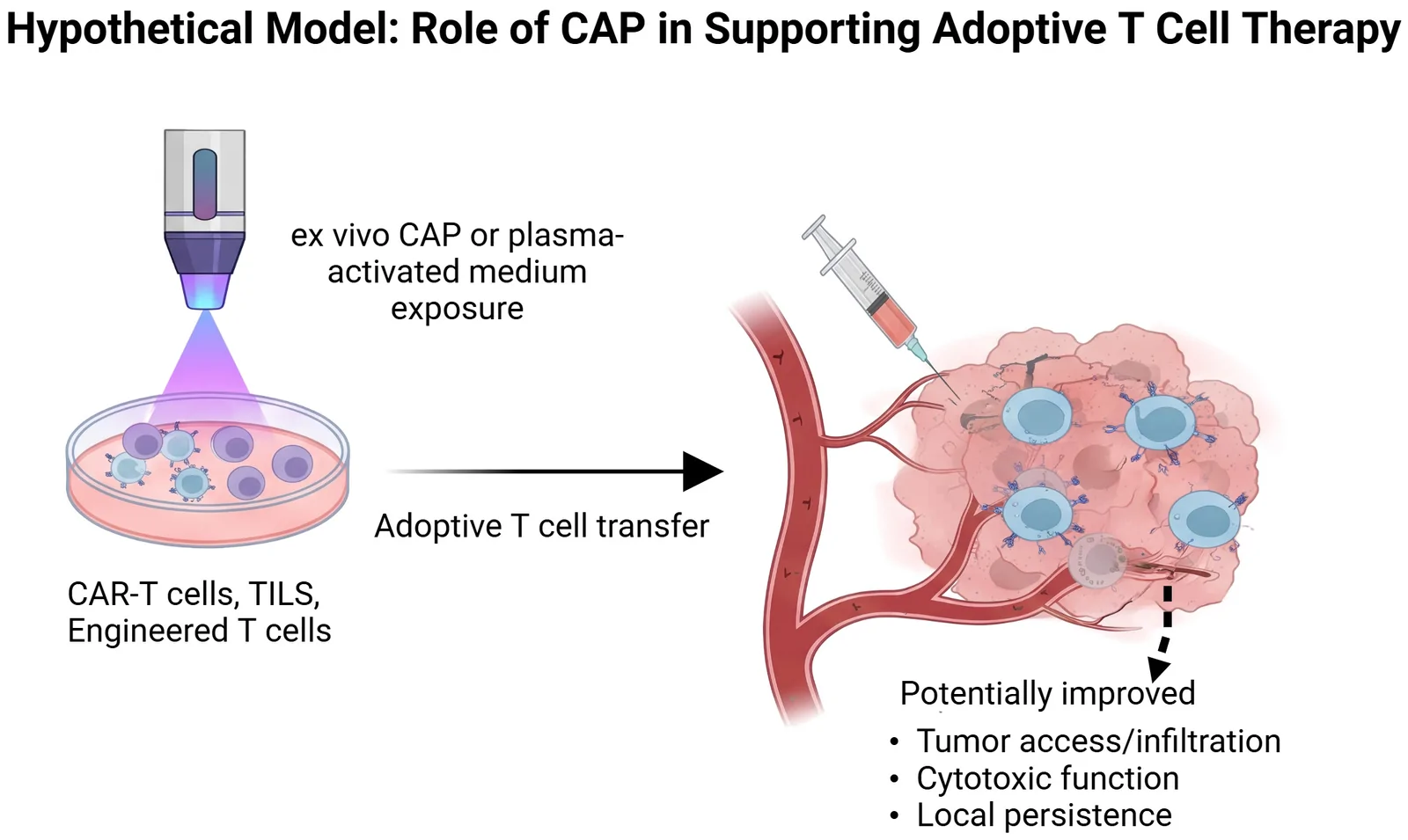
Hypothetical role of CAP in supporting adoptive T-cell therapy. Carefully controlled *ex vivo* exposure of CAR-T cells, tumor-infiltrating lymphocytes (TILs), or other engineered T-cell products to cold atmospheric plasma or plasma-activated medium/liquids has been proposed as a potential strategy to modify subsequent T-cell function. Possible downstream effects after adoptive transfer include improved tumor access or infiltration, cytotoxic function, and local persistence. However, direct CAP conditioning of therapeutic T-cell products remains hypothetical. The optimal exposure conditions and effects on T-cell viability, proliferation, metabolic fitness, exhaustion, memory differentiation, tumor specificity, and antitumor efficacy have not been established. Excessive CAP-derived RONS exposure may impair primary T-cell function or induce lymphocyte toxicity.

## Indirect T-cell activation through tumor and APC conditioning

The more strongly supported mechanism is indirect modulation of T-cell immunity through treatment of tumor cells/tissue, or using plasma-activated liquids. CAP can induce oxidative tumor cell stress and death (**Figure 3**), accompanied in some models by calreticulin exposure, ATP release, HMGB1 release, cytokine production, and other ICD-associated signals (**Figure 4**) (9, 10, 23, 24). These changes may facilitate APC recruitment, phagocytosis of tumor components, dendritic cell (DC) maturation, and cross-presentation of tumor-associated antigens. However, the presence of individual ICD-associated markers should not be interpreted as conclusive proof of functional ICD. Surface calreticulin, extracellular ATP, and HMGB1 release are biologically relevant danger signals, but they do not independently establish that tumor cell death generates durable, protective adaptive immunity. Stronger evidence includes DC activation with functional antigen presentation, vaccination strategies using CAP-treated tumor cells, tumor rechallenge experiments, and protection against antigen-matched or antigen-distinct tumor clones (25–28).

**Figure 3:**
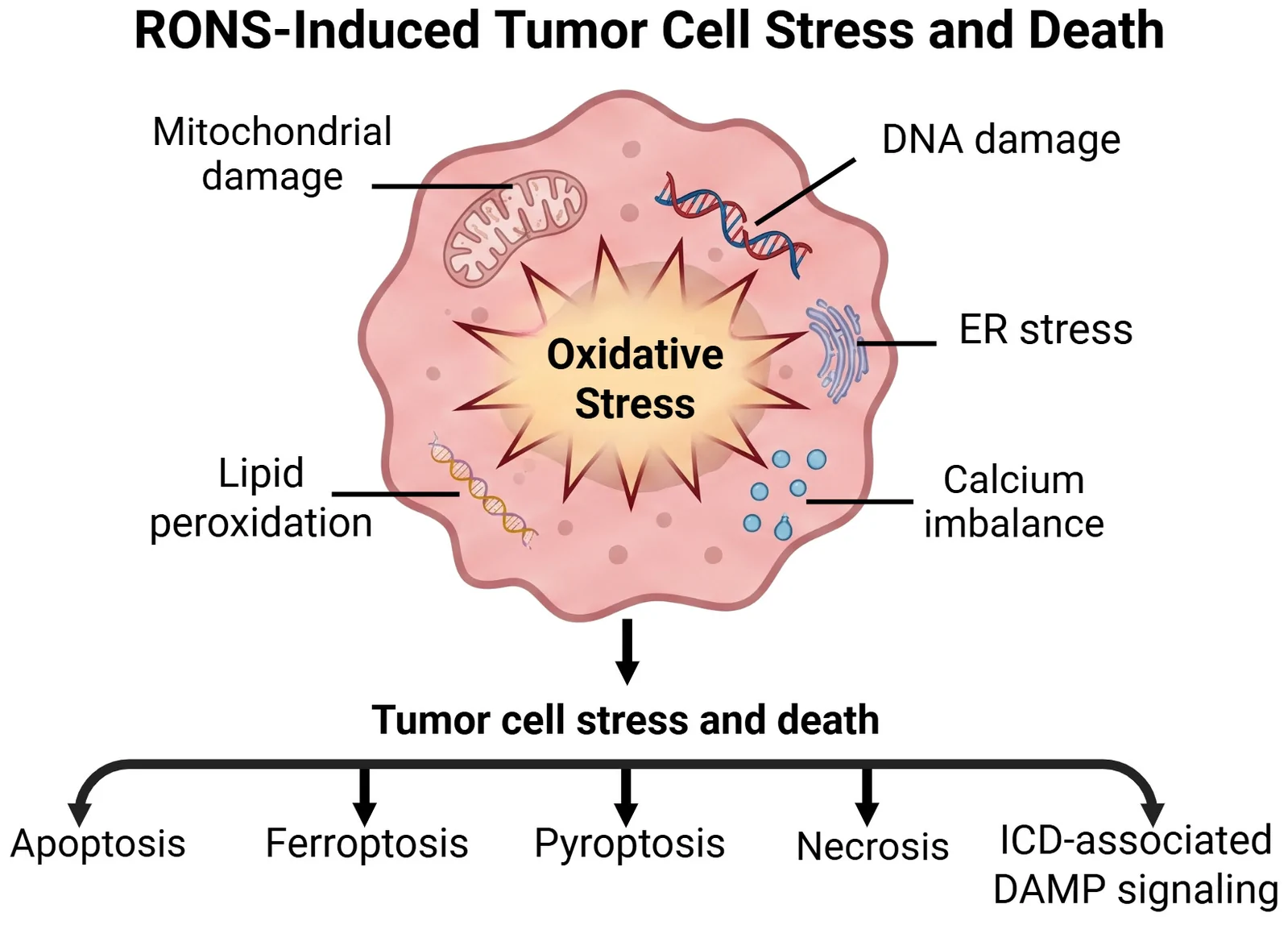
CAP-derived RONS can induce tumor cell stress and diverse cell-death pathways. CAP-induced oxidative stress may cause mitochondrial dysfunction, DNA damage, endoplasmic reticulum stress, calcium dysregulation, and lipid peroxidation. These stress responses can contribute to apoptosis, ferroptosis, pyroptosis, necrotic, and other context-dependent death programs. Under selected conditions, dying tumor cells may exhibit ICD-associated danger signaling.

**Figure 4:**
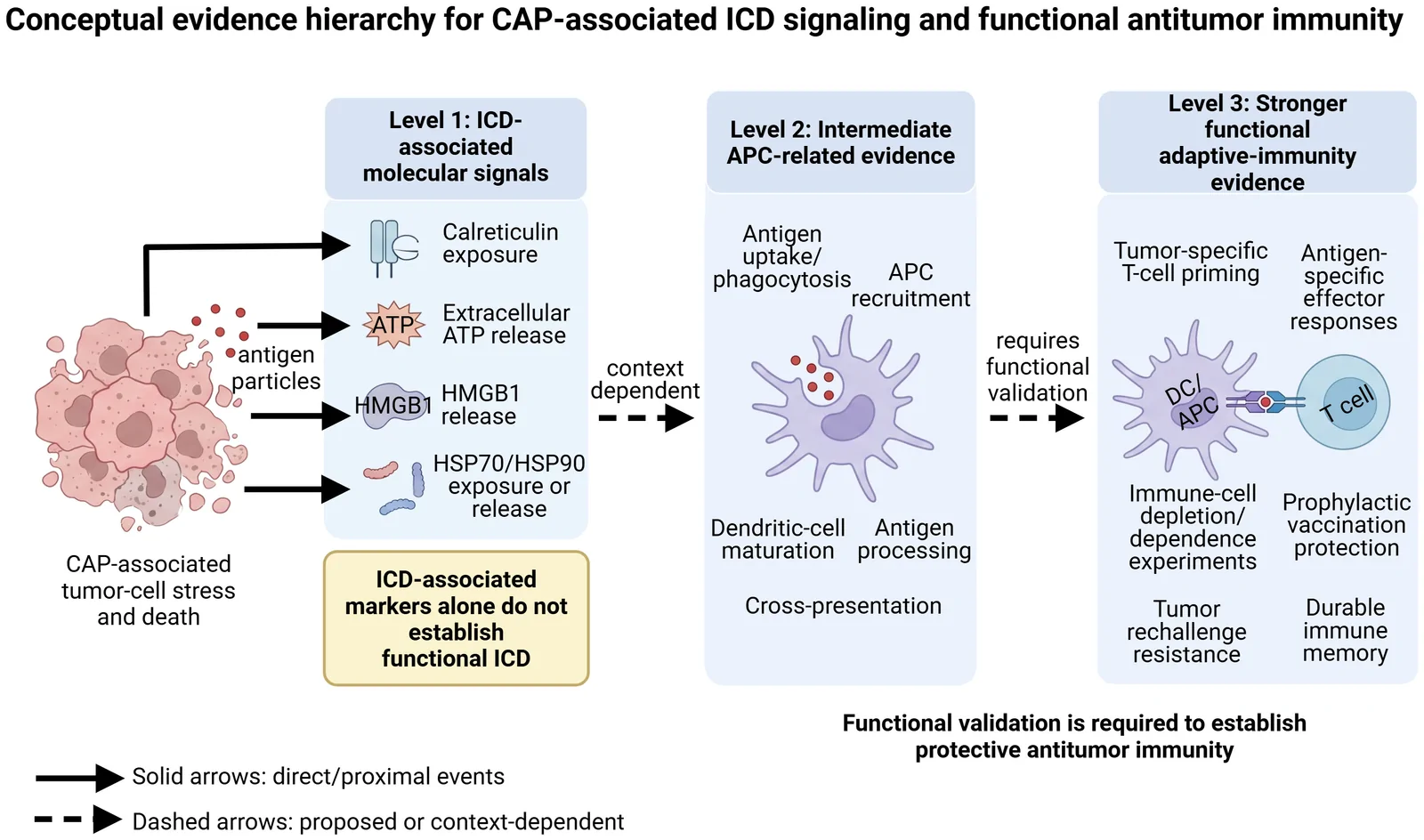
Conceptual evidence hierarchy for CAP-associated ICD signaling and functional antitumor immunity. CAP-induced oxidative stress and tumor cell death may be accompanied by the exposure or release of ICD-associated signals, including surface calreticulin exposure, extracellular ATP, HMGB1 release, and HSP70/HSP90 exposure or release. These markers indicate immunogenic stress but do not independently establish functional immunogenic cell death. Intermediate evidence includes APC recruitment, phagocytosis of tumor-derived material, dendritic cell maturation, antigen processing, and cross-presentation. Stronger functional evidence requires demonstration of tumor-specific T-cell priming and effector activity, immune-cell dependence, prophylactic vaccination protection, tumor rechallenge resistance, or evidence of durable immune memory. This figure is a conceptual evidence framework; solid arrows indicate reported proximal associations in selected experimental models, whereas dashed arrows indicate proposed or context-dependent downstream relationships requiring functional validation.

Several preclinical studies support this indirect T cell-activating role of CAP (**Tables 1**, **2**; **Supplementary Tables 1**, **2**). In murine B16F10 cultures, plasma-generated RONS induced tumor cell stress and death accompanied by ICD-associated signals, including reduced metabolic activity and migration together with extracellular release of ATP and CXCL1. Exposure of splenocytes to plasma-treated tumor cells or their conditioned supernatants triggered rapid Ca²^+^ influx and increased ERK1/2 phosphorylation and upregulation of the co-stimulatory receptor CD28 in CD3^+^ T cells, indicating early activation (29). In addition, a proinflammatory and T-cell supportive profile was observed, characterized by elevated levels of interleukin-1β (IL-1β), tumor necrosis factor-α (TNF-α), and CCL4 and changes in monocyte-associated markers. These mediators are known to reinforce effector CD8^+^ T-cell recruitment and function. Importantly, there was no increase in exhaustion markers such as PD-1 or TIM-3. These findings support the concept that CAP-treated tumor cells can generate immune-stimulatory signals; however, they do not by themselves demonstrate durable tumor-specific T-cell memory or direct CAP-mediated T-cell activation.

**Table 1:** Selected *in vitro*, *ex vivo*, and *in silico* studies relevant to CAP-mediated adaptive immune modulation.

Model/system	CAP treatment format	Principal immune-related finding	Evidence category	Key limitation	Reference
Mouse peritoneal macrophages and splenic T cells	Direct *ex vivo* helium CAP-jet treatment	CAP-treated macrophages showed increased IL-6 and IL-12 and reduced IL-10 and PD-L1; stimulated T cells showed increased IL-2 and IFN-γ production	Direct mixed immune-cell evidence	T-cell exposure conditions, viability, phenotype, antigen specificity, and long-term function were not adequately assessed	(18)
THP-1 monocytes, primary monocytes, and A375 melanoma co-cultures	Direct argon CAP treatment	CAP induced oxidative changes, altered monocyte-associated phenotypes, and increased inflammatory mediators; CAP-conditioned THP-1 cells enhanced short-term melanoma-cell killing	Myeloid/APC-related evidence	No APC antigen-presentation assay, T-cell co-culture, or tumor-specific adaptive-immunity endpoint	(78)
Primary human monocyte-derived dendritic cells	Direct argon CAP treatment	CAP increased DC maturation/co-stimulatory markers and cytokine release; DCs were more resistant than lymphocytes to CAP-associated toxicity	APC-related evidence	No antigen uptake, antigen presentation, DC–T-cell co-culture, or antigen-specific T-cell assay	(79)
Primary rat macrophages and T cells	Direct helium CAP-jet treatment	CAP-conditioned macrophages enhanced OVA peptide-stimulated OT-II CD4^+^ T-cell IL-2 and IFN-γ production	Antigen-dependent T cell-associated evidence through macrophage conditioning	Preprint; antigen presentation, T-cell viability, proliferation, memory, and long-term function were incompletely characterized	(80)
CAP-treated B16F10 melanoma cells co-cultured with murine splenocytes	Direct argon CAP treatment of tumor cells before co-culture	ATP and CXCL1 release; altered CD28 expression and myeloid cell-associated markers in splenocyte co-cultures	ICD-associated signals + T cell/myeloid-associated evidence	No antigen-specific priming, T cell-mediated tumor killing, or protective-immunity assay	(29)
Plasma-oxidized tumor proteins exposed to THP-1 cells and monocyte-derived DCs	CAP oxidation of proteins before APC exposure	Plasma-oxidized proteins promoted APC maturation and activation	APC-related evidence	Oxidative modifications and RONS were not comprehensively characterized; no cross-presentation or T-cell activation assay	(13)
*In silico* PD-1/PD-L1 and PD-1/PD-L2 molecular-dynamics models	Computational modeling of oxidation-associated modifications	Predicted destabilization of PD-1/PD-L1 and PD-1/PD-L2 interactions	Mechanistic hypothesis (*in silico*)	Requires biochemical and functional validation; modeled modifications may not reflect device-specific CAP chemistry	(39)
Experimental 3D tumor models and melanoma-bearing mice with CD47–SIRPα simulations	CAP treatment with complementary computational analysis	CAP reduced antibody-detectable CD47; simulations supported oxidation-associated weakening of CD47–SIRPα binding	Indirect innate-checkpoint/APC-related mechanism with *in silico* support	Reduced antibody binding does not independently prove CD47 downregulation or oxidation; phagocytosis, APC activation, tumor-specific T-cell priming, and durable adaptive immunity were not directly assessed	(38)

Evidence categories distinguish ICD-associated signals, APC-related evidence, T cell-associated evidence, functional protection/rechallenge evidence, and mechanistic hypotheses. Detailed CAP device parameters and treatment conditions are provided in **Supplementary Table S1**.

**Table 2:** Selected *in vivo* studies on CAP-associated immune modulation and adaptive antitumor immunity.

Tumor model/system	Treatment format	Principal immune-related outcome	Evidence category	Key limitation	Reference
CT26 colorectal carcinoma	Direct nanosecond-pulsed dielectric-barrier-discharge CAP treatment; vaccination-related experiments	ICD-associated signals, increased APC infiltration, and modest antigen-specific IFN-γ-producing T-cell responses	ICD-associated signals + APC recruitment + limited tumor-specific T-cell evidence	T-cell infiltration, exhaustion, memory, antigen specificity, and intratumoral physicochemical parameters were incompletely characterized	(26)
B16F10 melanoma rechallenge model	Direct nanosecond-pulsed humidified-oxygen streamer treatment	Delayed recurrent and distant tumor growth with increased CD8^+^ T-cell infiltration	Functional protection/rechallenge evidence compatible with adaptive immunity	Tumor-antigen specificity, TCR clonality, and the cellular mechanism of protection were not established	(27)
CT26 peritoneal carcinomatosis	Intraperitoneal plasma-treated saline	Increased macrophage abundance and CD25-associated CD4^+^ T-cell activation; reduced tumor burden	Indirect TME-conditioning and T cell-associated evidence	Limited T-cell functional, antigen-specific, exhaustion, and memory analyses	(23)
CT26 peritoneal carcinomatosis	Intraperitoneal plasma-oxidized Ringer’s lactate	Increased intratumoral CD4^+^ and CD8^+^ T cells and inflammatory cytokines; reduced tumor burden	T-cell infiltration- and cytokine-associated evidence	No antigen-specific T-cell analysis, cytotoxicity assay, or chemically matched reactive-species controls	(30)
B16F10 melanoma	Local CAP treatment with anti-PD-1	Increased immune-cell infiltration and improved tumor control in combination-treated animals	ICD-associated + APC-related + T cell-associated evidence with checkpoint-combination benefit	Optimal treatment sequence, T-cell function, antigen specificity, and predictive biomarkers were not established	(32)
B16F10-luc melanoma	Local argon CAP with systemic anti-PD-1	Increased DCs and macrophages, altered antigen-presentation/checkpoint signatures, and increased T-cell activation-associated markers	Preclinical combination-benefit evidence with TME immune remodeling	Tumor-antigen specificity, TCR clonality, cytotoxic function, and durable memory were not assessed	(33)
B16F10-fLuc melanoma	Transdermal helium CAP with local anti-PD-L1 microneedle delivery	Increased DC maturation, intratumoral CD4^+^/CD8^+^ T cells, Ki67^+^/granzyme-B^+^ CD8^+^ T-cell features, and control of local and distant tumors	Combination-benefit evidence with APC and T cell-associated outcomes	Single superficial melanoma model and limited follow-up; microneedle delivery may not be applicable to deep or internal tumors	(31)
SCC7 head-and-neck squamous-cell carcinoma	Direct ambient-air CAP with systemic anti-PD-1	Improved tumor control and survival with increased CD3^+^, CD4^+^, and CD8^+^ T-cell infiltration	T-cell infiltration evidence with checkpoint-combination benefit	Functional T cell, APC, Treg, exhaustion, and antigen-specific analyses were limited	(34)
Breast cancer and B16F10 melanoma	Intratumoral CAP-activated hydrogel with or without locally delivered anti-PD-1	CAP-gel induced ICD-associated signals and, with anti-PD-1, increased mature DCs, M1-like macrophages, CD4^+^/CD8^+^ T cells, and local/distant tumor control while reducing Tregs and MDSCs	ICD-associated + APC-related + T cell-associated evidence with checkpoint-combination benefit	Relative contributions of CAP chemistry, hydrogel carrier, and anti-PD-1 remain unresolved	(37)
CT26 colorectal carcinoma	Intratumoral CAP/trehalose hydrogel with or without local anti-PD-1	ICD-associated stress signals, DC/macrophage remodeling, increased T-cell infiltration, and improved combination-treatment outcomes	ICD-associated + APC-related + T-cell infiltration evidence with checkpoint-combination benefit	Tumor-antigen specificity, adaptive-immune dependence, and durable memory require further characterization	(53)
MX-7 murine rhabdomyosarcoma in C3H/He mice	Direct helium CAP-jet treatment	Increased serum HMGB1 after CAP exposure; limited cytokine changes in healthy mice	ICD-associated signal only; no functional adaptive-immunity evidence	Tumor growth, survival, intratumoral immune composition, APC activation, and T-cell function were not assessed; HMGB1 release alone does not establish functional ICD	(81)
Postsurgical B16F10 melanoma and 4T1 breast-cancer recurrence models	Local injection of CAP-DECM gel into the surgical cavity, with or without locally loaded anti-PD-L1	CAP-DECM gels increased ICD-associated signals, mature DCs, CD4^+^ and CD8^+^ T cells, and M1-like macrophage-associated responses; reduced postsurgical recurrence. CAP-DECM combined with anti-PD-L1 improved tumor-free survival and conferred resistance to B16F10 rechallenge	Functional protection/rechallenge evidence with ICD-associated, APC-related, and T cell-associated endpoints	The relative contributions of CAP-derived reactive species, DECM-associated chemokines/cytokines, tumor cell recruitment, local anti-PD-L1 delivery, and specific immune-cell subsets were not independently dissected; antigen-specific T-cell responses and depletion studies were not reported	(63)
Orthotopic MB49 bladder cancer in C57BL/6 mice; humanized bladder-cancer PDX model	Intravesical argon CAP treatment, alone or combined with systemic anti-PD-1	CAP reprogrammed tumor-associated neutrophils, reduced mitophagy, increased intratumoral CD8^+^ T-cell infiltration, and suppressed tumor growth. CAP plus anti-PD-1 produced greater tumor control and survival benefit than either monotherapy.	Myeloid-to-T-cell mechanistic evidence with adaptive-immune dependence and checkpoint-combination benefit	Tumor-antigen specificity, TCR clonality, durable memory, and the precise mechanisms linking neutrophil reprogramming to T-cell recruitment and function were not established	(57)
CAP treatment of normal skin in tumor-bearing mice	Nanosecond-pulsed humidified-oxygen streamer discharge applied to normal skin	Delayed distant-tumor growth in immunocompetent mice; effect absent in T cell-deficient and SCID mice	Hypothesis-generating evidence of a T cell-dependent abscopal-like effect	Treated-skin, draining-node, systemic, antigen-specific, and RONS-dosimetry analyses were limited	(82)
Prophylactic melanoma-vaccination model	Vaccination with plasma-oxidized tumor-associated proteins	Altered CD8^+^ T-cell activation, proliferation, and central-/effector-memory-associated phenotypes	Antigen-associated T-cell response evidence in a prophylactic setting	Established tumors were not treated; durable protection and antigen-specific TCR responses were not definitively shown	(14)

Evidence categories distinguish ICD-associated signals, APC-related evidence, T cell-associated evidence, functional protection/rechallenge evidence, and hypothesis-generating mechanistic observations. Detailed CAP device parameters, treatment schedules, and delivery formats are provided in **Supplementary Table S2**.

In CT26 colorectal cancer models, plasma-treated saline reduced tumor burden and was associated with changes in tumor immune infiltrates and *ex vivo* CD4^+^ T-cell responses (23). Similarly, gas plasma-treated Ringer’s lactate solution increased infiltration of both CD4^+^ and CD8^+^ T cells into CT26 tumors and elevated intratumoral levels of IL-2, interferon-γ (IFN-γ), and IL-6, indicating a more inflammatory microenvironment that may favor immune-cell recruitment and activation (30). These studies provide indirect preclinical evidence that CAP-treated liquids can condition the tumor environment in a manner compatible with enhanced adaptive immunity. Nevertheless, detailed analyses of T-cell clonality, antigen specificity, memory differentiation, exhaustion state, and long-term persistence remain limited.

## Antigen modification and T-cell priming

In addition to increasing antigen release, CAP may alter the antigenic properties of tumor-derived proteins through oxidation. CAP-induced protein oxidation has been reported to enhance human APC maturation and activation, suggesting a possible role in facilitating antigen processing or providing immunostimulatory signals (13). More recently, oxidized melanoma antigens were reported to promote activation and proliferation of cytotoxic T-cell subpopulations (14). These findings identify oxidative antigen modification as a potentially important mechanism linking CAP-derived RONS to adaptive immunity.

Nevertheless, it remains unclear whether CAP-induced protein oxidation generates modified peptide determinants that are processed, presented by APCs through MHC molecules, and specifically recognized by tumor-reactive T cells. Oxidation of tumor-associated proteins should not automatically be equated with neoepitope formation. Demonstration of a *bona fide* oxidative neoepitope would require identification of the modified peptide, evidence of MHC presentation, and selective recognition by antigen-specific T cells. Thus, CAP-induced protein oxidation is a promising mechanism that may alter antigen processing or immunogenicity, rather than established neoepitope formation.

## CAP and T-cell recruitment into tumors

CAP may influence T-cell recruitment indirectly through redox-sensitive regulation of cytokines, chemokines, cell stress pathways, and myeloid cell function. Such changes could potentially enhance effector-cell entry into tumors that are poorly infiltrated. However, the frequently used description of CAP as converting “cold” tumors into “hot” tumors remains a hypothesis supported by selected preclinical observations, rather than a broadly established conclusion. To establish cold-to-hot conversion, future studies should quantify not only total CD8^+^ and CD4^+^ T-cell density, but also spatial localization, tumor-reactive clonotypes, effector function, proliferation, memory differentiation, exhaustion markers, and interactions with APCs, tumor cells, endothelial cells, and stromal compartments.

## CAP in combination with immune checkpoint blockade

Several preclinical studies suggest that CAP may increase responsiveness to immune checkpoint blockade. In melanoma and other tumor models, CAP-mediated tumor cell stress, ICD-associated signaling, increased immune infiltration, and altered programmed death-ligand 1 (PD-L1) expression have been associated with improved tumor control when CAP was combined with anti-PD-1 or anti-PD-L1 therapy (31–34). In B16F10 melanoma models, local treatment with CAP or plasma-activated medium increased intratumoral CD4^+^ and CD8^+^ T-cell infiltration, supporting a potential combination benefit between CAP-based tumor conditioning and T cell-directed immunotherapy. CAP treatment was also associated with increased expression of IFN-γ, IL-6, IL-12p70, IL-2, TNF-α, and PD-L1 on residual tumor cells (31).

These findings provide a preclinical rationale for evaluating CAP-based tumor conditioning before or alongside checkpoint blockade. However, the effects of CAP on checkpoint pathways are complex and require careful interpretation. Increased PD-L1 expression is not a beneficial endpoint by itself and may reflect adaptive immune resistance driven by local inflammatory signaling, especially IFN-γ produced during antitumor immune responses (35, 36). In this setting, PD-L1 induction may provide a rationale for combining CAP with PD-1/PD-L1 blockade, but therapeutic benefit should be demonstrated by appropriate tumor control and functional immune endpoints rather than inferred from PD-L1 expression alone (31, 37). CAP may also influence checkpoint biology through oxidation-related changes in receptor–ligand interactions (38, 39). The biological and therapeutic consequences of such changes remain context-dependent and require validation using standardized models and clinically relevant dosing schedules. Evidence for CAP combined with CTLA-4, LAG-3, TIGIT, or other checkpoint inhibitors remains limited.

Overall, the current literature supports the conclusion that CAP can influence T-cell immunity mainly through tumor cell and TME conditioning, rather than through established direct T-cell activation. The most important next steps are device-specific dose–response studies, quantitative RONS measurements, functional antigen-presentation assays, and longitudinal analyses of tumor-reactive T-cell recruitment, differentiation, exhaustion, memory, and persistence.

## B cells and humoral immunity: limited and emerging evidence

Compared with T-cell responses, evidence concerning CAP-mediated B-cell modulation and humoral immunity remains very limited. Available studies primarily address B-cell viability and oxidative sensitivity rather than activation, antigen presentation, germinal-center responses, antibody production, class switching, or memory formation (19). Therefore, no firm conclusions can currently be drawn regarding whether CAP enhances or suppresses protective B-cell immunity.

Available evidence suggests that resting peripheral blood B cells are sensitive to CAP-derived oxidative stress. In comparative analyses of human blood mononuclear cell subsets, B cells showed reduced viability after direct plasma exposure, indicating that their tolerance threshold may be lower than that of several myeloid cell populations (19). However, susceptibility may differ according to B-cell maturation state, activation status, tissue origin, and exposure format. It is also unclear whether CAP-treated tumors alter regulatory B-cell populations or influence antibody responses against tumor-associated antigens. Future studies should assess B-cell viability, activation markers, antigen presentation, germinal-center formation, immunoglobulin isotypes, antibody specificity, plasma cell differentiation, and memory B-cell development. Until such studies are available, B-cell effects should be presented as an important knowledge gap rather than an established component of CAP-mediated adaptive immunomodulation.

## CAP-induced immunogenic cell death, DAMP signaling, and oxidative antigen modification

Triggering cancer cell death is an effective strategy to elicit antitumor immune responses. During this regulated form of cell death, dying cells expose or release damage-associated molecular patterns (DAMPs) that facilitate antigen uptake and immune activation (28). Among these, calreticulin is the best-characterized DAMPs, which translocate to the cell surface and flags dying cells for uptake by DCs, whereas HMGB1 release can activate TLR4-dependent signaling in APCs. Extracellular ATP can support recruitment and differentiation of DC precursors through purinergic signaling (40–42).

CAP-derived RONS may induce endoplasmic reticulum stress and activation of redox-sensitive integrated stress-response pathways, including PERK activation and downstream eIF2α phosphorylation (10, 43, 44). Concurrently, mitochondrial oxidative stress may trigger cytochrome c release and activation of the caspase-3/7 cascade, resulting in apoptotic cell death with an immunogenic phenotype (45–48). CAP may preferentially increase intracellular oxidative stress in malignant cells relative to non-malignant counterparts under selected experimental conditions; however, this selectivity is not universal and depends on cell type, cellular antioxidant capacity, plasma source, treatment parameters, and local microenvironmental conditions (49).

It is important to distinguish ICD-associated markers from functional immunogenicity. The presence of one or more of these signals alone does not establish that dying tumor cells induce protective adaptive antitumor immunity. Stronger evidence requires functional demonstration of DC activation, tumor antigen uptake and presentation, tumor-specific T-cell responses, vaccination effects, or protection against tumor re-exposure in appropriate rechallenge models. Accordingly, studies reporting only ICD-associated markers should be interpreted as evidence of immunogenic stress or a possible ICD phenotype rather than definitive proof of functionally ICD (26–28).

CAP may also influence adaptive immunity through oxidative modification of tumor-associated proteins and immune-regulatory surface molecules. Oxidized proteins may differ in their uptake and processing by APCs, interaction with innate sensors, and capacity to stimulate antigen-specific lymphocytes. Plasma-induced protein oxidation has been shown to promote maturation and activation of human APCs (13), while oxidized melanoma antigens can activate and expand cytotoxic T-cell subpopulations (14). These findings broaden the mechanistic framework beyond antigen release alone and suggest that CAP may alter both the quantity and immunological quality of tumor-associated antigens available for immune recognition. In addition, CAP-mediated oxidation may also affect innate immune-checkpoint signaling. In experimental three-dimensional (3D) tumor models and melanoma-bearing mice, CAP reduced antibody-detectable CD47 on cancer cells. Complementary molecular-dynamics and umbrella-sampling analyses predicted that oxidation-induced conformational changes in CD47 weaken its interaction with signal-regulatory protein alpha (SIRPα), a macrophage-associated inhibitory receptor. These findings suggest that CAP may reduce the CD47-mediated “don’t-eat-me” signal and thereby favor phagocytic clearance of stressed or dying tumor cells. Thus, CAP-derived RONS may potentially enhance immune recognition through two complementary mechanisms: increasing the immunogenicity of tumor-associated antigens and reducing inhibitory phagocytic signaling. Although experimental and computational evidence supports oxidation-associated weakening of the CD47–SIRPα interaction, the downstream contribution of this mechanism to macrophage phagocytosis, APC activation, tumor-specific T-cell priming, and durable antitumor immunity requires further clarification (38).

Preclinical studies indicate that direct CAP exposure or plasma-activated liquids can induce tumor cell cytotoxicity and ICD-associated signals in pancreatic cancer cells (**Tables 1**, **2**). In particular, CAP-treated phosphate-buffered saline or Ringer’s lactate induced tumor cell cytotoxicity together with ICD-associated molecular signals (50, 51). However, pancreatic tumors are characterized by dense stromal barriers, hypoxia, abnormal extracellular matrix (ECM) deposition, and complex interactions among tumor cells, stellate cells, endothelial cells, and immune cells. Therefore, findings from simplified *in vitro* systems should not be assumed to predict immune responses in pancreatic tumors *in vivo*.

Across mouse tumor models, CAP has reduced tumor burden and, in selected studies, prolonged survival (32, 52). These effects may result from direct cytotoxicity, immunogenic stress signaling, TME remodeling, and immune-mediated mechanisms, although their relative contribution remains uncertain.

CAP-based hydrogels combined with immune checkpoint inhibitors have generated local and systemic antitumor immune responses in several solid-tumor models, supporting further investigation of these approaches in pancreatic cancer (37, 53). However, clinical evidence for CAP-mediated adaptive immune modulation in pancreatic or other internal malignancies is still lacking.

## Tumor microenvironment remodeling and CAP-induced adaptive immunity

The TME is a critical determinant of whether local CAP treatment can generate meaningful adaptive antitumor immune responses. CAP acts within a complex environment comprising cancer-associated fibroblasts (CAFs), endothelial cells, ECM components, hypoxia, soluble mediators, extracellular vesicles, and infiltrating immune cells (**Figure 5**). These factors may influence RONS transport and persistence, redox buffering, tumor accessibility, immune-cell trafficking, and overall treatment responsiveness (54–56).

**Figure 5:**
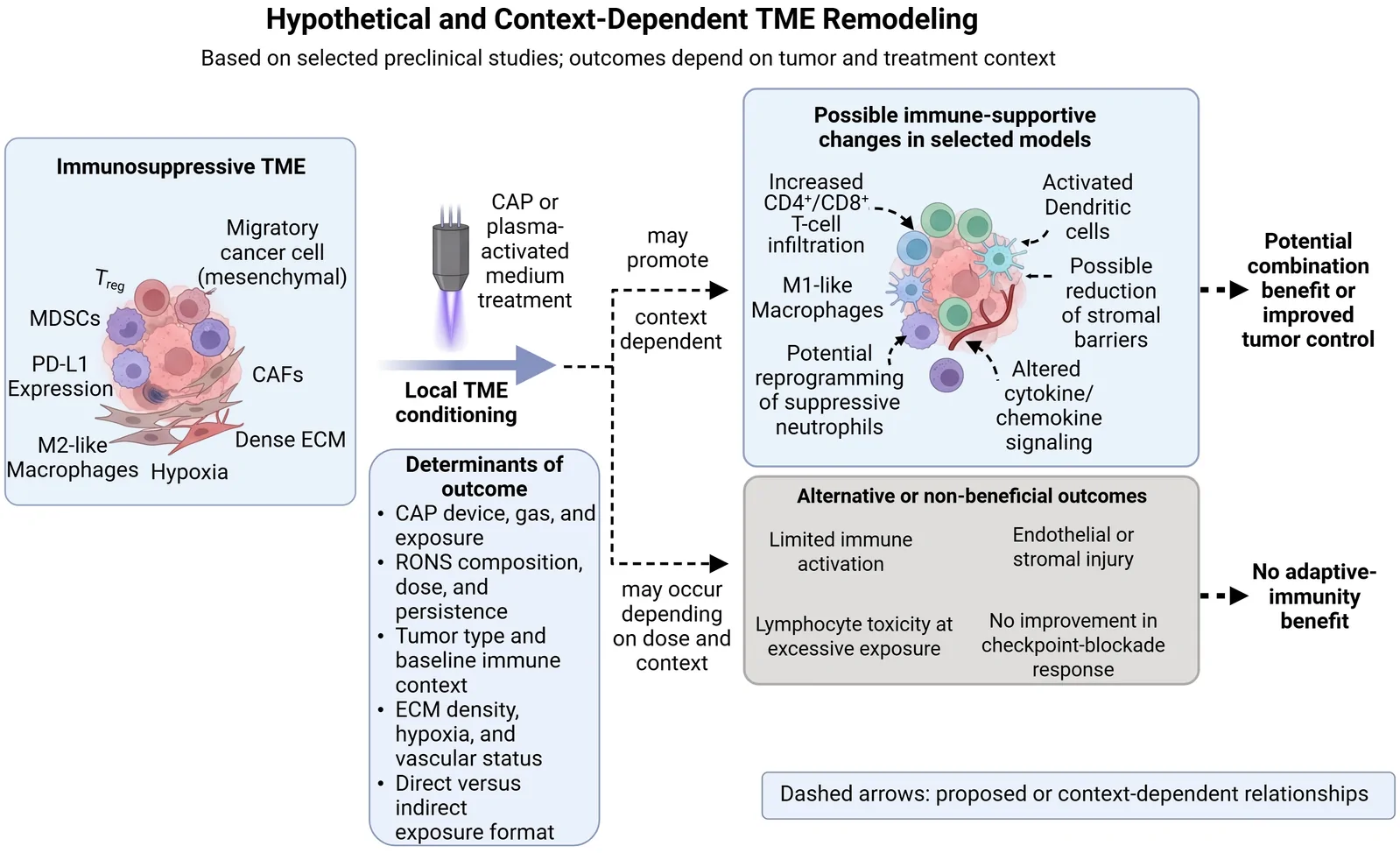
Hypothetical and context-dependent CAP-mediated remodeling of the tumor microenvironment. CAP or plasma-activated liquid treatment may alter tumor cell stress responses, cytokine and chemokine signaling, myeloid cell states, stromal barriers, vascular function, and immune-cell access. In selected preclinical models, these changes have been associated with increased infiltration of CD4^+^ and CD8^+^ T cells, activated dendritic cells, and M1-like macrophages. However, the effects of CAP on CAFs, extracellular matrix, hypoxia, vascular function, and other TME components remain incompletely characterized. Therefore, conversion of an immunosuppressive TME toward a more immune-active state and the resulting improvement in tumor control should be regarded as context-dependent preclinical possibilities rather than established universal outcomes.

The relevance of CAP-induced TME remodeling to adaptive immunity depends on whether changes in non-lymphoid compartments enhance tumor antigen uptake and presentation, facilitate recruitment of tumor-reactive T cells, or reduce local immunosuppressive and physical barriers that limit T-cell function (38, 57). Available preclinical studies indicate that CAP and plasma-activated liquids can modify tumor cell viability, cytokine and chemokine production, and intratumoral immune-cell infiltration (23, 29, 30, 58). In selected models, CAP treatment has been associated with increased intratumoral CD4^+^ and CD8^+^ T-cell infiltration, elevated inflammatory cytokine levels, and improved responses to anti-PD-1 or anti-PD-L1 therapy (30–32, 34). However, increased immune infiltration alone does not establish tumor-specific T-cell priming, effector function, or durable immune memory.

Myeloid cell remodeling may provide one mechanistic link between CAP exposure and adaptive immunity. CAP-associated modulation of macrophage phenotype or attenuation of CD47–SIRPα signaling may enhance phagocytic uptake of stressed tumor cells, thereby increasing the availability of tumor-derived material for APC processing and potential cross-presentation (38). More recent evidence provides a clearer link between CAP-mediated myeloid remodeling and adaptive immunity. In orthotopic bladder-cancer models, intravesical CAP inhibited neutrophil mitophagy and promoted an immunostimulatory neutrophil state, accompanied by increased intratumoral CD8^+^ and granzyme-B^+^ CD8^+^ T cells. Neutrophil-depletion, adoptive-transfer, and Rag1-deficient mouse experiments supported contributions from neutrophils and adaptive immunity to tumor control. CAP also provided combination benefit with anti-PD-1 blockade (57).

Nevertheless, it remains unclear whether neutrophil or macrophage reprogramming directly promotes presentation of defined tumor antigens, tumor-specific T-cell priming, or durable adaptive immunity. These relationships require mechanistic validation using myeloid cell depletion or blocking experiments, antigen-specific T-cell assays, and T-cell receptor repertoire analyses.

The stromal and vascular compartments of the TME may further regulate adaptive immune consequences of CAP treatment by controlling physical and biochemical barriers to RONS delivery and immune cell migration. CAFs can regulate CAP responsiveness through ECM deposition, cytokine secretion, and stromal remodeling (56, 59). In selected experimental models, CAP has been reported to increase p53 signaling in CAFs, promoting apoptosis or senescence, and to inhibit differentiation of mesenchymal stem cells into CAFs, potentially reducing stromal support for tumor progression (44, 55, 56). CAP has also been reported to disrupt tumor endothelial integrity and attenuate microcirculation in solid tumors (60). These stromal and vascular effects may influence RONS penetration, vascular permeability, immune-cell trafficking, and treatment sensitivity. However, direct evidence linking CAP-mediated modulation of CAFs or endothelial cells to enhanced antigen presentation, T-cell priming, lymphocyte infiltration, or systemic antitumor immunity remains limited. In addition, extracellular vesicles released from CAP-treated tumor or stromal cells may potentially transfer oxidized biomolecules or immunoregulatory mediators to neighboring or distant cells, but this mechanism remains hypothetical in the context of CAP-mediated adaptive immunity.

Overall, CAP may condition the TME through coordinated effects on tumor cells, myeloid populations, stromal components, and endothelial cells. However, DAMP release, cytokine production, stromal changes, or increased immune-cell infiltration should not be interpreted as proof of tumor-specific adaptive immunity. CAP-induced effects are likely to depend on CAP dose, device configuration, treatment timing, tumor type, and the pre-existing stromal and immune composition of the TME (10–12). Future studies should employ 3D tumor models, organoids, tumor explants, stromal-immune co-cultures, and immunocompetent orthotopic models. Spatial transcriptomics, multiplex imaging, and single-cell profiling will be important for defining how CAP modifies interactions among tumor, stromal, vascular, and immune-cell compartments.

## Cross-priming, epitope spreading, and immune-checkpoint modulation

CAP-induced tumor cell stress may support cross-priming by increasing the availability of tumor-derived material for uptake by APCs. In other ICD-inducing treatment settings, extracellular ATP can support the recruitment and differentiation of DC precursors through purinergic signaling, whereas chemokines such as CXCL10 can facilitate CXCR3-dependent recruitment of effector T cells to tumors (61, 62). Whether CAP consistently induces these pathways, recruits CD103^+^ cross-presenting DCs, and enhances cross-presentation of defined tumor antigens remains insufficiently demonstrated.

Vaccination and rechallenge studies using CAP-treated tumor cells have provided functional evidence of protective antitumor immunity in selected models (26, 27, 47). These findings are compatible with the induction of tumor-reactive adaptive immunity and may involve epitope spreading. However, such vaccination models do not necessarily demonstrate that local treatment of established tumors with CAP induces the same immune effects. Moreover, protection in challenge or rechallenge experiments does not by itself establish broadened antigen recognition, tumor-specific T-cell memory, or protection against antigenically distinct tumor clones. These questions require validation using immune cell depletion experiments, T-cell receptor sequencing, antigen-specific tetramers, and adoptive transfer approaches. Transdermal CAP-mediated delivery strategies combined with anti-PD-L1 have induced local tumor cell death and increased APC activation and cytotoxic T-cell activation, resulting in improved control of primary and distant tumors in mouse models (31).

CAP combined with anti-PD-1 has also shown enhanced tumor control and increased intratumoral CD4^+^ and CD8^+^ T-cell infiltration in selected melanoma and head-and-neck cancer models (32–34). Injectable plasma-activated hydrogels and trehalose-containing CAP-based hydrogels have further been reported to promote adaptive immune activation and improve responses to checkpoint blockade (37, 53).

More recently, an injectable CAP-activated decellularized extracellular matrix (CAP-DECM) gel was evaluated in postsurgical B16F10 melanoma and 4T1 breast-cancer models. CAP-DECM gel treatment was associated with ICD-related signals, DC maturation, increased local CD4^+^ and CD8^+^ T-cell responses, macrophage remodeling, reduced tumor recurrence, and systemic immune-associated changes. Local application of anti-PD-L1 further improved tumor-free survival, and mice cured by the combination treatment showed resistance to B16F10 tumor rechallenge (63).

These findings should nevertheless be interpreted as preclinical combination evidence. They do not yet establish optimal sequencing, device settings, treatment frequency, pharmacokinetic interactions, predictive biomarkers, or clinical benefit in patients. In addition, the relationship between CAP and PD-L1 biology is complex: CAP may influence checkpoint biology through multiple, potentially opposing mechanisms. Depending on the model and treatment conditions, CAP has been associated with altered PD-L1 expression, redox-dependent changes in checkpoint-protein structure, and inflammatory cytokine production (32, 39). The net consequence for checkpoint-blockade sensitivity remains context-dependent and requires further functional validation.

## Therapeutic applications of CAP-driven immunomodulation in the context of radiotherapy and chemotherapy

The combination of CAP with conventional radiotherapy and chemotherapy has gained considerable attention, driven by the hypothesis that CAP-derived RONS can potentiate tumor cell killing while simultaneously amplifying antitumor immune responses (10, 44). By delivering tunable RONS directly to the tumor, CAP exploits cancer cell redox vulnerability and may sensitize tumor cells to drugs and ionizing radiation, offering a rationale for multimodal treatment strategies (64). In melanoma, CAP is primarily studied pre-clinically in combination with chemotherapy and, more recently, radiotherapy (43, 65). For an adaptive-immunity-focused framework, the relevant question is whether these combinations not only increase tumor cell killing but also improve antigen release, APC activation, T-cell recruitment, or response to immunotherapy.

In B16F10 melanoma cells, CAP combined with ionizing radiation increased apoptosis and cell-cycle arrest *in vitro* (65). Notably, plasma pre-treatment sensitized tumor cells to subsequent radiation rather than *vice versa*. Supernatants from co-treated tumor cells contained elevated levels of TNF-α, IL-6, and granulocyte-macrophage colony-stimulating factor (GM-CSF). When applied to the murine DC line JAWS, these supernatants induced MHC-II and CD83, alongside increased expression of PD-L1 and PD-L2, suggesting that combined treatment may influence immunogenic signaling. However, these experiments were performed *in vitro* and do not establish that CAP–radiotherapy combinations generate tumor-specific T-cell responses *in vivo*.

In combination with chemotherapy, CAP has been paired with canonical ICD-inducing agents including oxaliplatin, doxorubicin, and dacarbazine (43, 66–68). CAP exposure of various melanoma cell lines (B16F0, B16F1, and SK-MEL28) followed by treatment with ICD-inducing agents increased the release of ATP and CXCL10 from melanoma cells. This, in turn, promoted THP-1 monocyte chemotaxis toward the TME and augmented ICD (43, 66). CAP alone, and especially in combination with the chemotherapeutic agent dacarbazine, reduced melanoma growth more effectively in a preclinical model (68). Thus, it appears that CAP may have the potential to eliminate melanoma cells and enhance the efficacy of chemotherapy, which might improve tumor control in the future. A recent systematic review and meta-analysis of 41 studies reported enhanced effects of CAP and doxorubicin on melanoma cell viability and cytotoxicity, although effects on specific cell death endpoints such as apoptosis induction showed less consistency across studies, and the evidence remains predominantly *in vitro* (67).

Thus, CAP–radiotherapy and CAP–chemotherapy combinations are promising primarily as preclinical tumor-conditioning strategies. Their capacity to improve adaptive antitumor immunity remains plausible but incompletely demonstrated. Future studies should incorporate immunocompetent models, APC and T-cell functional assays, tumor rechallenge experiments, and analyses of treatment sequencing. It will be particularly important to determine whether CAP should be administered before, during, or after chemo- or radiotherapy to maximize immunogenic tumor cell death while minimizing damage to infiltrating lymphocytes.

From a translational perspective, CAP-based chemo- and radiotherapy combinations may increase tumor cell susceptibility to conventional treatment and potentially enhance ICD-associated signaling. Whether these effects translate into improved tumor-specific adaptive immunity, reduced systemic toxicity, or clinically meaningful dose reduction remains to be demonstrated.

## Physicochemical controls for interpreting CAP-mediated immune effects

Interpretation of CAP-mediated immune effects requires controls that separate plasma-derived chemistry from nonspecific effects of gas flow, handling, heating, evaporation, and changes in the liquid microenvironment. CAP is a multicomponent stimulus comprising RONS, electric fields, charged particles, photons, and local thermal and fluid-dynamic effects, and the balance among these components depends strongly on device architecture, gas composition, flow rate, treatment distance, voltage, and exposure time (11, 69, 70). Depending on the plasma source and experimental design, appropriate controls should include sham treatment, gas-flow-only exposure, and monitoring or matching of temperature, pH, liquid volume, evaporation, and medium-handling time. For direct CAP exposure, the possible contributions of electric fields, charged particles, UV radiation, and local gas-flow effects should be considered. These considerations are particularly important during direct CAP exposure, in which cells may be exposed to combined chemical and physical plasma components, including short-lived reactive species and physical components that are absent, transformed, or substantially attenuated in plasma-activated liquids (55, 71).

For plasma-activated liquids, mechanistic attribution should begin with quantitative characterization of commonly measured long-lived species, particularly hydrogen peroxide (H_2_O_2_), nitrite (NO_2_^−^), and nitrate (NO_3_^−^), and should extend, where feasible, to secondary oxidation products generated through reactions with medium components. This is important because plasma-treated liquids are chemically dynamic and matrix-dependent systems whose composition is influenced by buffering capacity, ionic strength, antioxidants, amino acids, serum components, liquid volume, treatment geometry, and post-treatment aging (72, 73). Chemically matched control solutions containing H_2_O_2_, nitrite, nitrate, or defined combinations of these species can help determine whether selected stable species reproduce particular biological effects (74). Such controls should also be matched for pH, osmolality, ionic composition, and, where relevant, solvent and buffer composition.

However, such controls cannot fully reproduce the complex, device-specific, and time-dependent chemistry of plasma-treated liquids, including short-lived reactive species, secondary reactions with medium components, and oxidation products of proteins or lipids. Therefore, attribution of immune effects to individual RONS should be supported by quantitative chemical measurements and functional validation rather than inferred from CAP exposure alone.

## Clinical translation, dose heterogeneity, and knowledge gaps

The clinical translation of CAP for cancer immunotherapy remains at an early stage. The interpretation and translation of CAP-based cancer immunotherapy require consideration of the effective RONS exposure, delivery format, target cell susceptibility, strength of available evidence, and major translational barriers (**Figure 6**). CAP has been used clinically in wound care and dermatological settings, where studies have demonstrated feasibility, local tolerability, and antimicrobial or wound-healing effects (75, 76). These clinical data are valuable for establishing aspects of device safety and local tissue compatibility. However, they do not demonstrate clinical antitumor efficacy, systemic immune activation, or therapeutic benefit in oncology.

**Figure 6:**
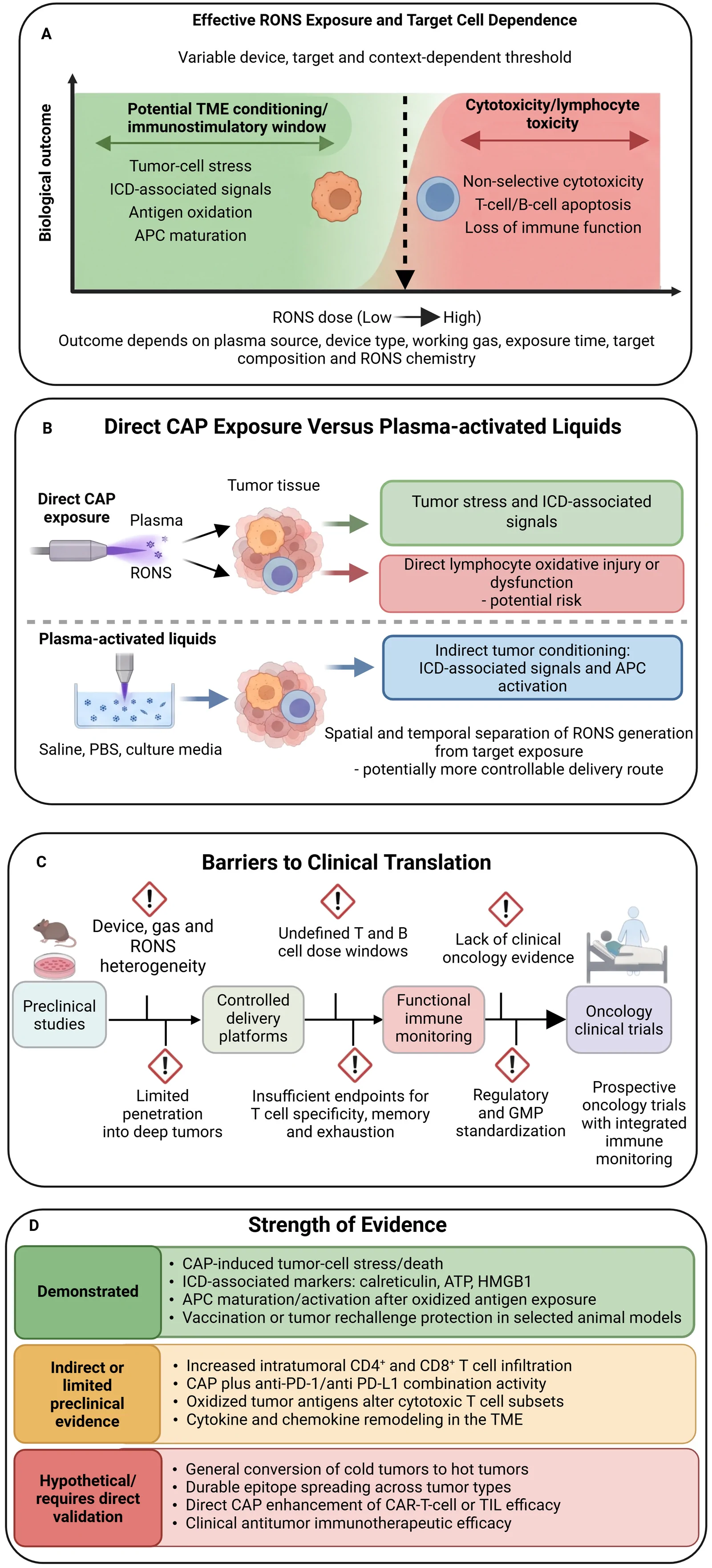
Critical framework for CAP-mediated adaptive immunomodulation and clinical translation. **(A)** Biological effects of CAP depend on the effective RONS exposure, target cell susceptibility, and local biological context. Lower or intermediate exposures may induce tumor cell stress, ICD-associated danger signaling, oxidative antigen modification, and APC activation, whereas excessive or poorly controlled exposure may cause non-selective cytotoxicity, lymphocyte dysfunction, or apoptosis. The transition between these effects is device-, target-, and context-dependent. **(B)** Direct CAP treatment and plasma-activated liquids differ in RONS generation, delivery, persistence, and the potential for direct lymphocyte exposure. Direct CAP treatment may induce tumor cell stress and ICD-associated signals in selected preclinical models but may also cause oxidative lymphocyte injury. Plasma-activated liquids provide spatial and temporal separation between plasma generation and administration and may enable indirect tumor conditioning; however, their chemical composition, bio-distribution, and immunological effects remain context-dependent. **(C)** Translation requires robust immunocompetent preclinical models, standardized device-specific dosimetry, reproducible delivery platforms, functional immune monitoring, regulatory and GMP-compliant development, and prospective oncology trials with integrated immune endpoints. **(D)** Current evidence includes demonstrated preclinical findings, indirect or limited preclinical observations, and proposed mechanisms or applications requiring direct functional validation. In particular, durable tumor-specific T-cell memory, general cold-to-hot tumor conversion, improved CAR-T cell or TIL efficacy, direct B-cell activation, and clinical antitumor immunotherapeutic efficacy remain insufficiently established.

At present, evidence for CAP-mediated adaptive antitumor immunity is predominantly derived from *in vitro* systems and murine models. Therefore, claims that CAP can convert immunologically “cold” tumors into “hot” tumors, induce durable T-cell memory, or provide clinically meaningful benefit when combined with approved immune checkpoint inhibitors should be interpreted cautiously until validated in well-designed clinical trials (**Figure 6**).

A major translational barrier is that “CAP treatment” does not represent a single standardized intervention. Biological outcomes depend on multiple variables, including plasma source, device configuration, working gas, voltage or power, frequency, treatment distance, exposure duration, target geometry, tissue composition, liquid volume, buffer composition, and RONS stability (10–12). These parameters determine the composition, concentration, and persistence of short- and long-lived RONS, as well as the contribution of other plasma-associated factors such as electric fields, photons, local pH changes, temperature, and secondary liquid-phase chemistry. As a result, CAP exposure can produce divergent biological outcomes, including tumor cell death, inflammatory activation, immunoregulation, or lymphocyte toxicity.

This dose dependence is especially important for adaptive immunity. Low or appropriately localized RONS exposure may promote tumor cell stress, antigen release, ICD, and APC activation. In contrast, excessive or poorly controlled exposure may impair the viability and function of primary T cells, B cells, or therapeutic immune-cell products (19–22). Accordingly, future studies should report device-specific parameters and, wherever possible, include quantitative measurements of relevant RONS, pH, temperature, and other physicochemical characteristics. Reporting treatment time alone is insufficient, because identical treatment durations can generate markedly different chemical and biological effects across plasma devices and experimental systems.

Several key biological and translational questions remain unresolved:

- It remains unclear whether CAP enhances tumor-specific T-cell priming, clonal expansion, cytotoxicity, memory differentiation, persistence, and trafficking, or whether its effects are primarily limited to early activation markers and cytokine production.
- The effects of CAP treatment intensity, timing, and repetition on exhaustion markers, metabolic fitness, transcriptional and epigenetic states, and effector-memory differentiation remain poorly defined.
- The ability of CAP-treated tumor cells or plasma-activated liquids to enhance B-cell antigen presentation, antibody responses, or humoral antitumor immunity requires further investigation, particularly in relation to B-cell viability, tolerance, and regulatory function.
- How do fibroblasts, endothelial cells, ECM, hypoxia, extracellular vesicles, and tumor metabolism influence RONS transport and immune outcomes?
- CAP may be valuable in combination with anti-PD-1, anti-PD-L1, chemotherapy, radiotherapy, therapeutic antibodies, nano-adjuvant platforms, CAR-T cells, or tumor-infiltrating lymphocyte therapy (31, 37). However, optimal sequencing, dose scheduling, treatment intervals, and patient-selection criteria remain undefined.
- The consequences of repeated CAP treatment require systematic evaluation, including effects on genomic integrity, local tissue injury, autoimmunity, tumor evolution, off-target immune-cell damage, and oxidative injury to therapeutic T cells during *ex vivo* or *in vivo* combination strategies (10, 31, 37, 77).
- How can CAP or plasma-activated liquids be delivered to deep-seated tumors while maintaining reproducible chemistry and minimizing normal tissue exposure?
- Reproducibility remains limited by variation in plasma devices, exposure parameters, experimental models, and RONS quantification. Standardized reporting frameworks, validated dosimetry, and harmonized immune assays are essential. Regulatory pathways will also be required for device certification, manufacturing standardization, and good manufacturing practice (GMP)-compliant development of CAP-based combination products.
- Experimental controls and mechanistic attribution: Future studies should include sham-treatment and gas-flow-only controls, together with monitoring or matching of temperature, pH, liquid volume, evaporation, osmotic conditions, and medium-handling time. Chemically matched H_2_O_2_/nitrite/nitrate conditions should also be used where appropriate. These measures are necessary to distinguish RONS-mediated immune effects from other physical or chemical features of plasma treatment.

For clinical translation, future trials should include immune-monitoring endpoints in addition to conventional tumor-response measures. These should assess tumor immune-cell composition, CD8^+^ and CD4^+^ T-cell localization, APC activation, cytokine and chemokine profiles, T-cell receptor clonality, circulating immune changes, oxidative stress, and local tissue injury biomarkers. Such studies will be necessary to determine whether CAP produces clinically meaningful adaptive immune modulation rather than local cytotoxicity alone.

## Emerging future directions

Several research priorities may advance CAP from a promising local redox intervention to a more rigorously defined immunotherapeutic platform.

CAP-mediated tumor conditioning should be investigated as a potential strategy before or alongside established immunotherapies. For example, CAP-mediated remodeling of the TME before CAR-T cell or TIL infusion is a hypothesis that warrants investigation. It may improve tumor accessibility, antigen availability, or local immune conditions; however, effects on T-cell engraftment, tumor infiltration, persistence, metabolic fitness, and antitumor activity have not yet been established.

Future studies should also use multiparametric immune endpoints. These should include antigen-specific T-cell responses, T-cell receptor repertoire analysis, memory and exhaustion phenotyping, spatial distribution of immune cells, and functional assessment of APC–T cell interactions. Single-cell RNA sequencing, ATAC sequencing, proteomics, spatial transcriptomics, and multiplex imaging may identify predictive biomarkers, and define responder versus non-responder immune profiles.

Experimental studies should incorporate standardized plasma characterization. At a minimum, investigators should report plasma source, gas composition, electrical settings, treatment geometry, exposure duration, liquid composition, and available measurements of RONS, pH, and temperature. This information is necessary to compare studies and identify dose windows that favor tumor immunogenicity over lymphocyte toxicity.

Prospective clinical studies should distinguish local safety, tumor cytotoxicity, and immunological efficacy. Early oncology trials should be designed with translational immune endpoints and should evaluate CAP as part of rational combination regimens rather than assuming that local CAP treatment alone will generate systemic antitumor immunity.

Finally, progress across all domains will ultimately depend on interdisciplinary collaboration between plasma physicists, tumor immunologists, clinical oncologists, and regulatory scientists, working toward protocols that are mechanistic, reproducible, scalable, and safe.

## Conclusions

The immunomodulatory potential of CAP is promising but remains insufficiently mature for clinical implementation in T cell-based cancer immunotherapy. Current evidence supports CAP primarily as a preclinical tumor-conditioning strategy that may indirectly enhance adaptive antitumor immunity. The best-supported mechanistic rationale is indirect: CAP can induce tumor cell stress and, in selected models, cell death accompanied by ICD-associated danger signals; it may also modify tumor-associated antigens, condition APCs, and reshape local immune and stromal signals. These effects may facilitate antigen presentation, T-cell priming, tumor infiltration, and responsiveness to immunotherapy, especially in CAP-conditioned combination approaches.

In contrast, direct enhancement of primary T-cell function by CAP remains insufficiently demonstrated and should be interpreted cautiously because lymphocytes are highly sensitive to oxidative stress. This is especially relevant for adoptive cell therapies, including CAR-T cells and tumor-infiltrating lymphocytes, where excessive CAP-derived RONS could impair T-cell viability, metabolic fitness, cytotoxicity, persistence, or memory differentiation. Therefore, CAP may be more rationally developed as a tumor- or microenvironment-conditioning intervention before adoptive T-cell transfer, rather than as a direct stimulant of therapeutic T cells. Whether CAP-mediated remodeling can improve CAR-T or TIL engraftment, trafficking, tumor penetration, and long-term persistence remains an important but still largely hypothetical area of investigation. Evidence for CAP-induced B-cell modulation and humoral antitumor immunity is even more limited. Similarly, claims that CAP induces epitope spreading, converts immunologically “cold” tumors into “hot” tumors, enhances CAR-T/TIL efficacy, or generates systemic antitumor immunity should currently be regarded as early preclinical possibilities rather than established outcomes.

Progress toward clinical translation will require standardized device-specific dosimetry, rigorous characterization of RONS and other physicochemical components, functional evidence of antigen presentation and tumor-specific T-cell responses, careful assessment of lymphocyte toxicity, and clinical trials incorporating immune-monitoring endpoints. If these requirements are met, CAP may become a useful local adjunct to increase tumor immunogenicity and improve selected T cell-based and adoptive immunotherapy strategies.

## Supplementary material

Supplementary Table 1 Detailed physicochemical and experimental characteristics of selected *in vitro*, *ex vivo*, and *in silico* studies relevant to CAP-mediated adaptive immune modulation.

Supplementary Table 2 Detailed physicochemical and treatment characteristics of selected *in vivo* studies on CAP-associated immune modulation and adaptive antitumor immunity.
