# Supplementary material for: Cold atmospheric plasma and adaptive antitumor immunity: current evidence with emphasis on T-cell responses
Source: Front Immunol. 2026 Oct 28;17:1935730. doi: 10.3389/fimmu.2026.1935730 (PMC13752760; doi:10.3389/fimmu.2026.1935730)
Supplement: Supplementary file 1 [file Table1.pdf]

Supplementary Table S1

Detailed physicochemical and experimental characteristics of selected *in vitro*, *ex vivo*, and *in silico* studies relevant to CAP-mediated adaptive immune modulation

**Model/system**	**CAP modality**	**Reported treatment parameters**	**Treatment format**	**Ref.**
Mouse peritoneal macrophages and splenic T cells	Direct helium CAP jet	Macrophages: 15 or 30 s; T cell exposure duration not reported	Direct *ex vivo* treatment of immune-cell populations	(18)
THP-1 monocytes, primary monocytes, and A375 melanoma co-cultures	kINPen atmospheric-pressure plasma jet	Argon, 2 standard L/min; THP-1 cells: 20 or 120 s; primary monocytes: 10 or 20 s	Direct treatment of monocytes before phenotypic and co-culture analyses	(78)
Primary human monocyte-derived dendritic cells	kINPen atmospheric-pressure plasma jet	Argon, 2 standard L/min; approximately 1 MHz; approximately 1 W; 15-mm nozzle-to-liquid distance; 60, 120, or 180 s	Direct treatment of monocyte-derived dendritic cells	(79)
Primary rat macrophages and T cells; OVA peptide-stimulated OT-II T cells	Direct helium CAP jet	Helium, 1.5 L/min; 3 kV; 12.5 kHz; macrophages: 15–30 s; T cells: 60–120 s; jet-tip temperature <40 °C	Direct immune-cell treatment and macrophage-mediated assessment of antigen-stimulated T cell responses	(80)
B16F10 melanoma cells and murine splenocytes	kINPen atmospheric-pressure plasma jet	Argon, 3 standard L/min; 2–6 kV peak-to-peak; approximately 1 MHz; 20-mm nozzle-to-well distance; 120 s for co-culture experiments and 180 s for migration assays	Direct treatment of tumor cells before splenocyte co-culture	(29)
Plasma-oxidized tumor proteins exposed to THP-1 cells and monocyte-derived dendritic cells	kINPen atmospheric-pressure plasma jet	Tumor proteins in PBS; 5-mm nozzle-to-liquid distance; 60 s treatment	Indirect exposure of APCs to plasma-oxidized proteins	(13)
In silico PD-1/PD-L1 and PD-1/PD-L2 complexes	Molecular-dynamics simulations	Selected oxidation-associated modifications introduced computationally	Computational modeling only	(39)
U87 glioblastoma and SC263 head-	Microsecond-pulsed dielectric-barrier-	DBD output: 30 kV; 1–1.5 µs rise time; 2 µs pulse	Direct CAP treatment of 3D tumor models and	(38)

Model/system	CAP modality	Reported treatment parameters	Treatment format	Ref.
and-neck squamous-cell-carcinoma spheroids; A375 melanoma in ovo tumors; B16F10 melanoma-bearing C57BL/6 mice; CD47–SIRPα molecular-dynamics simulations	discharge (DBD) plasma	width. Spheroids: 500 Hz for 10 s, electrode positioned 1–2 mm above cells. *In ovo* A375 tumors: 700 Hz for 10 s. B16F10 tumors: 700 Hz for 10 s, device positioned approximately 1 mm above tumor	tumors; B16F10 tumors were treated once daily for 5 consecutive days. Molecular-dynamics and umbrella-sampling simulations assessed effects of modeled CD47 oxidation on CD47–SIRPα binding	

Treatment parameters are reported as described in the cited publications. Direct comparisons among studies are limited by differences in plasma source, treatment geometry, target composition, and biological models.

Supplementary Table S2

Detailed physicochemical and treatment characteristics of selected *in vivo* studies on CAP-associated immune modulation and adaptive antitumor immunity

Tumor model/host	CAP modality	Reported treatment parameters	Treatment route/schedule	Ref.
CT26 colorectal carcinoma in mice	Nanosecond-pulsed dielectric-barrier discharge	*In vitro*: 10 s, 29 kV, 30 Hz, 1-mm discharge gap. *In vivo*: 10 s, 29 kV, 750 Hz; 3-mm electrode positioned approximately 1 mm above the tumor	Direct local tumor treatment once daily for 5 days; additional vaccination-related experiments	(26)
B16F10 melanoma rechallenge model	Nanosecond-pulsed humidified-oxygen streamer discharge	Humidified oxygen, 0.5 L/min; 26 kV; 8-ns pulse duration; 100 pulses/s; electrode positioned 5 mm above tumor	Direct tumor treatment for 10 min/day for 5 consecutive days	(27)
CT26 peritoneal carcinomatosis	kINPen plasma-treated saline	Argon, 5 standard L/min; 20- or 60-min saline treatment	Intraperitoneal administration of 300 µL untreated or plasma-treated 0.9% NaCl for 5 days	(23)
CT26 peritoneal carcinomatosis	kINPen plasma-oxidized Ringer's lactate	Argon, 3 standard L/min; 1 MHz; plasma source positioned 1 cm above liquid surface; 20-min liquid treatment	Intraperitoneal administration every second day for five cycles	(30)
B16F10 melanoma	Microsecond-pulsed dielectric-barrier-discharge (DBD) plasma	30 kV; 700 Hz; 1–1.5 µs pulse rise time; 2 µs pulse width; 0.9 mJ/pulse; total energy approximately 6.3 J per treatment; applicator positioned 1–3 mm above the tumor	Direct local treatment for 10 s once daily for 5 consecutive days, beginning 3 days after tumor inoculation when tumors were palpable	(32)
B16F10-luc melanoma in C57BL/6 mice	kINPen argon plasma jet with anti-PD-1	Argon, 3 standard L/min; approximately 1 MHz; approximately 1 W; 5-min local CAP treatment	Three local CAP-treatment cycles combined with intraperitoneal anti-PD-1	(33)
B16F10-fLuc melanoma	Transdermal helium CAP with anti-PD-L1	Approximately 11 kV; 12 kHz; helium, 16.5 L/min; 4-min CAP treatment	Local anti-PD-L1 delivery using a hollow microneedle patch	(31)

Tumor model/host	CAP modality	Reported treatment parameters	Treatment route/schedule	Ref.
	microneedle delivery		containing 200 µg antibody	
SCC7 head-and-neck tumors in immunocompetent mice; pharyngeal xenografts in nude mice	Piezobrush PZ2 handheld piezoelectric direct-discharge CAP device	50 kHz; 0.9 mW; pharyngeal tumors received 1-min CAP treatment	CAP administered three times weekly; SCC7 tumors combined with intraperitoneal anti-PD-1 (200 µg)	(34)
Breast cancer in BALB/c mice and B16F10 melanoma in C57BL/6 mice	Needle–ring electrode CAP jet; helium-fed	Approximately 6 kV; helium flow rate, 8.5 L/min. One milliliter of deionized water, positioned 1 cm below the CAP jet, was treated for 1–10 min; CAP-treated water was used to prepare 20 wt% Pluronic F127 hydrogel	Intratumoral injection of 100 µL every 3 days for six doses	(37)
CT26 colorectal carcinoma in BALB/c mice	Needle–ring electrode CAP jet; helium-fed	Helium CAP-treated water: approximately 6 kV, 3.5 A, 8.5 L/min helium; 10-min water activation; incorporated into 20% Pluronic F127 hydrogel	Intratumoral gel injection every 2 days for six doses. For checkpoint-combination studies, anti-PD-1 was co-loaded into CAP/trehalose gel; treatment was administered every 2 days for six injections	(53)
MX-7 murine rhabdomyosarcoma in female C3H/He mice	Direct helium CAP jet	Helium, 9 L/min; 4.9 kV; approximately 40 kHz; approximately 7 mA; 1-min treatment; 2-cm treatment distance	Direct local treatment in two treatment courses	(81)
Postsurgical B16F10 melanoma/C57BL/6 and 4T1 breast-cancer/BALB/c models	Needle–ring CAP jet	Argon, 8.5 L/min; approximately 6 kV. DECM solution was CAP treated for 20 min before gel formation; 1 mL liquid positioned 1 cm below the jet	100 µL gel injected locally into the surgical cavity immediately after resection. Anti-PD-L1-containing CAP-DECM gel used 6 mg/kg anti-PD-L1. Cured mice were	(63)

Tumor model/host	CAP modality	Reported treatment parameters	Treatment route/schedule	Ref.
			rechallenged on day 50 with B16F10 cells	
Orthotopic MB49 bladder cancer in female C57BL/6 mice; humanized patient-derived bladder-cancer xenografts in hu-HSC-NOG-EXL mice	Double-layer dielectric-barrier-discharge argon plasma jet. In vivo delivery used a custom intravesical CAP sprayer with a flexible PTFE catheter	Argon, 18 L/h per tube; 15 kV; 1 kHz. Each intravesical CAP session lasted 15 min. Device temperature remained below 30°C during 15-min operation	Intravesical CAP (15 min) was administered every 5 days from day 5 after tumor inoculation; anti-PD-1 (10 mg/kg, intraperitoneal) was given every 3 days in combination studies. Humanized PDX mice received CAP every 5 days from day 7 after implantation.	(57)
Colorectal carcinoma in BALB/c, SCID, and BALB/c nu/nu mice	Nanosecond-pulsed humidified-oxygen streamer discharge applied to normal skin	Humidified oxygen, 0.5 L/min; 25 kV; 20-ns pulses; 100 pulses/s	Normal dorsal or abdominal skin treated for 10 min/day for 5 days	(82)
Prophylactic melanoma-vaccination model	Plasma oxidation of tumor-associated proteins using kINPen	Helium/2% oxygen, 1 standard L/min; protein concentration 100 µg/mL; 1-min treatment; >2-mm nozzle-to-liquid distance	Intraperitoneal vaccination with 10 µg plasma-oxidized protein followed by a booster dose after 7 days	(14)

Treatment parameters are reported as described in the cited publications. Biological effects should not be compared directly across studies because devices, gases, exposure geometries, treatment schedules, tumor models, and delivery formats differ substantially.
